# Professional Development among Secondary Teachers in Spain: Key Associated Factors as of PISA 2018

**DOI:** 10.3390/jintelligence11050093

**Published:** 2023-05-14

**Authors:** Juan Pablo Hernández-Ramos, Fernando Martínez-Abad

**Affiliations:** University Institute of Educational Sciences, University of Salamanca, 37008 Salamanca, Spain

**Keywords:** professional teacher development, smart schools, decision trees, educational data mining, education quality

## Abstract

Professional development for teachers is fundamental in the configuration and functioning of smart schools. This paper aims to characterize professional development with the participation of compulsory secondary teachers in Spain and to detect key factors in the functioning and organization of schools associated with higher levels of ongoing teacher training. A cross-cutting non-experimental design was used to conduct a secondary analysis of data from PISA 2018 tests, including over 20,000 teachers and more than 1000 schools in Spain. Descriptive results show great variability in teachers’ commitment to their professional development; this variability is not associated with the grouping of teachers by school. The decision tree model completed with data mining tools shows that intensive professional teacher development in schools is associated with a better school climate and higher levels of innovation, cooperation, taking on shared goals and responsibilities, and leadership distributed among the education community. The conclusions highlight the importance of ongoing teacher training and how this improves educational quality in schools.

## 1. Introduction

As with other individuals, teachers develop and learn based on their interactions with a series of school contexts or structures of a diverse nature and scope (Bronfenbrenner 1979). Teacher development associated with education quality must therefore be accompanied by favourable factors in terms of organisation, social structure, etc. In this context, professional teacher development is a fundamental element that provides teachers with tools to leverage those internal and external factors in order to promote educational excellence. We could say that education systems that achieve high levels of teacher training and professional development promote the development of high levels of corporate intelligence in schools (Bolivar 2013).

The Organisation for Economic Cooperation and Development (OECD), a body created to foster and develop policies that improve the economic and social welfare of people, focuses on the analysis and promotion of education quality as one of its key objectives. In this line, after analysing the different educational policies, this institution highlights the importance of considering schools as institutions capable of their evolution and development (OECD 2019b). This ability lies directly with the main educational agents in schools: teachers, who, with their training and development as education professionals, enable schools to improve and adapt to new social needs. Teacher education models, due to both the needs of teachers and the political, social, economic and cultural transformations that have taken place, have undergone countless changes in recent years (Lorenzo-Vicente et al. 2015). One of the most relevant issues is the formative nature of primary and secondary education, where certain doubts arise as to whether a general formative profile should be consolidated or give way to a more specialist one (Imbernón 2014); this is a vitally important aspect of initial teacher training (Hernández-Ramos 2021). Based on the latest report of the European Education and Culture Executive Agency et al. (2022), it is worth noting that teacher education should include both a general and a professional component. The first one is based on generic activities and subject specialisation, while the second one develops courses focused on the acquisition of skills characteristic of the teaching profession and practical training in schools. The simultaneity or achievement of this type of training continues to be a matter of study at present where both visions coexist (Lorenzo-Vicente et al. 2015), though a less relevant aspect in a study such as the present one where attendance at training activities is analysed without assessing the subject matter.

One of the OECD’s main activities in promoting improved education quality is the Programme for International Student Assessment (PISA). PISA is a large-scale international assessment carried out every three years in secondary education since 2000 (OECD 2018). This programme provides the academic and education community with a wide variety of educational data and indicators on national education systems in participating countries. The essential blocks of PISA include the following: student skill indicators; non-cognitive student traits; school organisation and climate; management team leadership; school resources; socio-economic, cultural and demographic factors of schools, students and teachers; and teaching practice quality. Of all large-scale international assessments, PISA is the most widespread globally at both an academic level (Avvisati 2020; Hernández-Torrano and Courtney 2021; Hopfenbeck et al. 2018) and in terms of dissemination (Jerrim 2023).

Despite its wide academic relevance and diffusion, the procedures and implications of large-scale assessments such as PISA have received some significant criticism. These appreciations are fundamentally focused on the process of construction and adaptation of the context questionnaires (Fernandez-Cano 2016; Gamazo and Martínez-Abad 2020; González-Such et al. 2016; Hopfenbeck et al. 2018; Jornet 2016; Rutkowski and Rutkowski 2010):

- Differences between countries in social, cultural and economic significance of the include constructs;
- Lack of temporal stability in the definition of the constructs and items included in different waves;
- Poor translation of the items into non-English languages;
- High rate of missing data in items and constructs.

Therefore, as Jornet (2016) points out, we must be cautious in the interpretations made based on results from large-scale international assessments. Understanding school improvement as a series of planned actions organised by schools to assume, prevent and solve problems (OECD 2019b), as well as to seek quality in teaching (Hernández-Ramos et al. 2021), this study focuses on detecting factors associated with teacher training so as to identify whether their professional development boosts smart schools, i.e., institutions that facilitate the development of all members of the education community. This diagnostic assessment will be carried out with information on schools, teachers and students provided in the PISA 2018 assessment (OECD 2019a).

Many researchers have analysed aspects directly linked with school improvement (Aderet-German and Ben-Peretz 2020; Bolivar et al. 2017; García-Martínez et al. 2018; Hargreaves 2003; Malagón and Graell 2022; Murillo and Krichesky 2012; Ortega-Rodríguez and Pozuelos 2022; Prenger et al. 2021). A literature review of these studies can identify three key factors related to aspects that can be improved by ongoing teacher training: (I) distributed and shared leadership; (II) collaboration culture and school climate; (III) innovation and development of quality educational practices. These factors are a conceptual framework of reference for this study.

Another key area of professional teacher development widely present in the literature is related to educational assessment (Aderet-German and Ben-Peretz 2020; Malagón and Graell 2022; Murillo and Krichesky 2012; Prenger et al. 2021). Despite its presence in academic literature and its importance for improving teaching-learning processes, an educational assessment must be omitted from this study as PISA tests do not include specific factors that can used as a reliable indicator of teachers using initial, formative and summative assessment.

### 1.1. Educational Leadership

Distributed leadership entails changes in the distribution of power, control and authority—traditional views of hierarchical leadership in schools—giving way to a micro-political perspective where a great number of teachers are involved in school organisation and management (García-Martínez et al. 2018). Authors such as Hallinger (2018) believe that the change in school leadership can be understood as a response to the changing needs of schools in the context of global education reforms. The leader traditionally offers a reward to those who obey an order; with distributed leadership, there are no orders or rewards, but coordination and mutual benefit. Distributed leadership, based on interaction and cooperation among all parties, is therefore established in groups in which various people share responsibilities for guiding and completing tasks (García-Martínez et al. 2018). In addition to emphasising the need to abandon old hierarchical models of leadership that are no longer valid and to develop new leaders at all levels of the system, Harris (2008) establishes four basic requirements for distributed leadership:

1. Have multiple levels of participation in decision making;
2. Focus on improving teaching practice and the real problems of the education community;
3. Consider all members of the group;
4. Be flexible.

Under this perspective, leading consists of preparing and empowering others, creating a collective responsibility with the goal of improving together (Martínez-Valdivia et al. 2022). Thus, shared and cooperative leadership style is an essential factor in improving education (Bolivar et al. 2017; Hallinger 2018; Villa 2019) and in creating smart schools.

The development of distributed school leadership is consistent with a collective vision of teachers as members of a school community who share a vision of the future, common concerns and values to promote.

### 1.2. Collaboration Culture and School Climate

Shared leadership style is closely linked with the second factor to be highlighted: collaboration culture and school climate. García-Martínez et al. (2018) emphasise the importance of factors such as teacher collaboration, greater flexibility of organisational structures or empowering teachers as a way to boost the professional capital of teachers by means of ongoing training. Working in collaborative contexts, with mutual support and shared responsibilities, generates a suitable work climate to address, solve and prevent any kind of circumstance. In smart schools, where teachers and students develop simultaneously, it is relevant that teachers form part of learning and collaboration communities both inside and outside the school. After analysing the benefits of collaboration among the school education community—also known as network governance—, authors such as Ehren and Perryman (2018) highlight that teachers involved in these networks show greater development of different key competencies, improving their knowledge, skills and attitudes toward teaching. A system of interconnected schools can advance together in the search of knowledge by exchanging resources, conveying information and adopting educational proposals that improve them as a whole (Robinson et al. 2020). With a climate of collaboration, work relationships between teachers are spontaneous, voluntary, unpredictable and geared towards the common development of both teachers and the school (Hargreaves 2003; Ortega-Rodríguez and Pozuelos 2022; Prenger et al. 2021).

### 1.3. Educational Innovation

Focusing on the third and final factor, educational innovation and the development of quality educational practices, we must note that these concepts are closely linked. An appropriate culture of collaboration is fundamental for innovation projects to become good practices (Hernández Ramos and Torrijos 2020). Innovation plays a relevant role in school improvement by incorporating different strategies and techniques, such as teacher research action (Ortega-Rodríguez and Pozuelos 2022), project-based learning (Toledo and Sánchez-García 2018), or the use of different technology resources (Fernández-Cruz and Fernández-Díaz 2016; Hernández-Ramos et al. 2021). Smart schools favour and foster the development of innovation projects that promote social justice and collaborative construction of knowledge between teachers and students. A good example are all the projects implemented in recent years under the service-learning philosophy, a vision of education that combines learning with social commitment (Marquez-García et al. 2022; Martínez-Valdivia et al. 2022).

Therefore, considering that the factors detailed above can and must be promoted and developed through teacher training, which is undoubtedly the most powerful tool in fostering educational improvement (Hernández-Ramos et al. 2022; Imbernón 2014; Malagón and Graell 2022), this paper focuses on analysing professional teacher development in Spanish compulsory secondary education teachers, and it characterises schools where teachers are more concerned with training and developing as education professionals. Thus, after an initial analysis of the distribution of ongoing training for secondary teachers in Spain, the study will focus on identifying the main factors related to leadership, collaboration culture and educational innovation emerging in schools with high levels of professional teacher development.

## 2. Materials and Methods

Based on the literature review and taking in account the fundamental goal of the paper, the following research questions (RQ) are proposed:

RQ1. What topics are secondary teachers in Spain most interested in for their professional development?
RQ2. How involved are secondary teachers in Spain in their professional development?
RQ3. Is a high level of professional teacher development associated with the promotion of smart schools (innovation in teaching practices, with the development of shared leadership styles and with an institutional culture of collaboration)?

This research is based on an analysis of secondary data from the large-scale PISA 2018 assessment. Therefore, in accordance with the PISA 2018 technical report (OECD 2017), a cross-cutting non-experimental research design was developed. This research is thus ex post factor, with exploratory and correlational interest.

Starting with a population of secondary school teachers in Spain, the PISA 2018 tests obtain a sample of 21,621 teachers in 1089 centres. An average of almost 20 compulsory secondary education (ESO in Spanish) teachers surveyed per school were included in the sample, with a standard deviation (Sx) = 4.08. The OECD applies a two-stage conglomerate stratified, probabilistic sampling in the PISA assessments, sampling schools in the first stage and education agents available in the centres (students and teachers) in the second.

The study criterion variable was obtained by adding the items TC045Q1-18, related to ongoing training activities teachers declare they have carried out in the last 12 months, resulting in a variable with a maximum score of 18 (the teacher states they have carried out training activities in the last 12 months related to all the topics proposed) and a minimum score of 0 (the teacher indicates they have not carried out any training activity in the topics indicated). At a teacher level, this variable can therefore be associated with the number of ongoing training activities by each teacher. At school level, this variable can be considered as the average volume of ongoing training activities carried out by teachers at the school. It is important to note that this variable was obtained from the response of teachers included in PISA on their participation in training activities, not from more objective sources of information such as management teams or the educational administration.

The explanatory variables included in this study are listed in Table 1. Given that the unit of analysis was schools, schools of interest variables (composite factors^1^ and demographic variables) and variables of interest at student (available composite factors) and teacher levels (available composite factors and variables related to ongoing training) were added to the schools database. As recommended in the PISA technical reports for cases in which it is not possible to work with the 10 available plausible values (OECD 2009), a single plausible value was selected at random (specifically number 10) in the student performance variables in mathematics (PV10MATH), performance in reading (PV10READ) and performance in science (PV10 SCIE).

Various general and specialised statistics software programs were used to carry out the data analysis. Firstly, since the JASP software does not include a module for editing the data matrix, the database was pre-processed with SPSS V.26. Specifically: (1) we obtained the study’s criterion variable by summing the variables TC045Q1-TC045Q18, in which teachers indicate whether he/she has carried out training activities in various subjects in the last 12 months; and (2) we aggregated to the school database the average of the explanatory variables of the student and teacher levels. Then, descriptive and inferential statistics were analysed with Microsoft Excel (frequency diagrams) and Jasp 0.16.4 (descriptive and inferential statistics and box plots). Finally, decision trees were applied with specialised data mining software Weka 3.8.5. Specifically, the J48 data mining algorithm (an extension of C4.5) was applied, recommended for obtaining simple models that can be interpreted at an applied level (Martínez-Abad and Chaparro Caso López 2017; Martínez-Abad et al. 2020). Missing values in the decision tree statistical model were processed with a probabilistic approach different from traditional imputation (Gamazo and Martínez-Abad 2020; Witten et al. 2016). Under this approach, cases with missing values are distributed in a weight manner among the tree branches in the same proportion as the percentage of subjects with observed values. These cases contributed to the fit of the predictive model in the same way as subjects without missing values. The use of decision trees is widespread in educational research when the number of explanatory variables available is large. In these cases, decision trees make it possible to obtain a simple and easily interpretable predictive model. Unlike regression models, these techniques do not present parsimony problems when there are many predictor variables, since they make a prior selection of predictor variables included in the model.

## 3. Results

### 3.1. Initial Exploration

**Figure 1:**
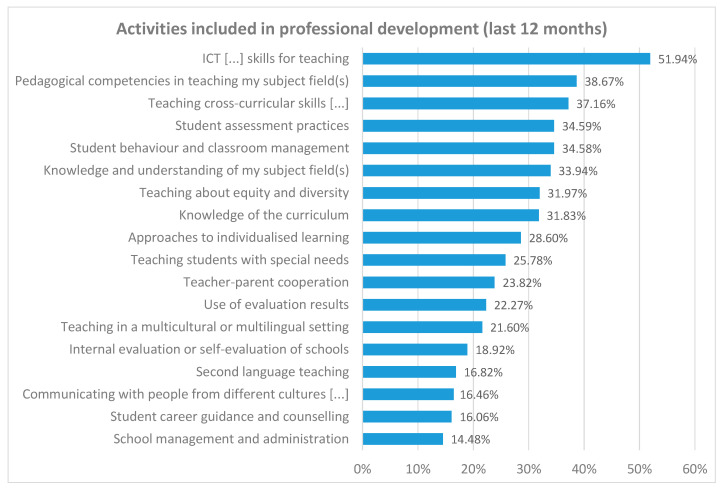
Professional development activity frequency.

First, we will analyse professional development activities completed more or less frequently by Spanish secondary teachers. Figure 1 shows these overall results. More than 50% of teachers say they have completed ICT activities to develop skills for teaching-learning.

With an interest in training of almost 40%, far from the interest shown in ICTs, are activities on specific pedagogical skills regarding teaching the teacher’s subject field (38.67%) and teaching cross-curricular skills (37.16%). At the other end of the scale, less than 20% of teachers state that, in the last 12 months, they have completed ongoing training activities related to school management and administration (14.48%), student career guidance and counselling (16.46%), effective communication with people from different cultures (16.46%), second language teaching (16.82%), and institutional school evaluation strategies (18.92%).

Table 2 shows the number of training activities teachers state they have completed in the last 12 months.

In the teacher database, the average value is slightly different under five activities, with a very high variable placing the coefficient of variation (*CV*) at practically 100%.

$$CV=\frac{S_{x}}{\bar{X}}=\frac{4.892}{4.995}=.9794$$

This means that, in average terms, the number of activities a teacher says they have completed deviates from the mean almost as much as it does from the average. A detail confirmed by observing the three quartiles: while at least 25% of teachers state they have completed professional training courses in only one of the topics available, another 25% state they have completed eight or more types of activities. This excessive variability means that there is a significant proportion of teachers who are barely involved in professional development in the Spanish education system, although it is also true that another good proportion are firmly committed to it.

This variable falls significantly when analysing data at school level, which means that the uneven commitment to professional development observed at teacher level is not replicated in schools. In fact, by calculating the intraclass correlation coefficient (*ICC*), which indicates the proportion of total variance of the number of training activities completed by the teacher explained by grouping teachers in schools, this value is lower than 4%:

$$ICC_{Teacher\;Development}\;=\frac{S_{interschool}^{2}}{S_{interschool}^{2}+S_{intraschool}^{2}}=\frac{0.9358}{0.9358+23.0035}=.0391$$

By dividing the total variance of a variable (in this case, the amount of teacher training activities) into the variability between subjects within groups, in this case schools ($S_{intraschool}^{2}$), plus the variability between groups ($S_{interschool}^{2}$), the *ICC* statistic indicates what proportion of the total variability of the amount of teacher training activities is due to differences between schools ($S_{interschool}^{2}$).

Therefore, with these data, we can confirm that, in Spain, there are both teachers highly involved and little involved in their professional development (great variability at teacher level); however, these differences in level of commitment are not explained by grouping teachers in different schools.

**Figure 2:**
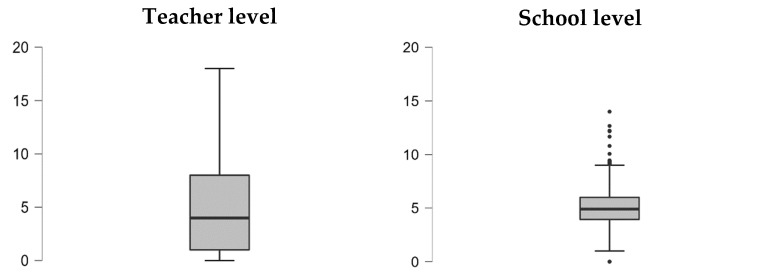
Box plot of the number of professional development activities, comparative between teacher level and school level.

Figure 2 shows the great variability in the number of training activities at teacher level, with a clear, positive asymmetric distribution, and how variability at school level is significantly reduced.

Table 3 presents a hypothesis contrast at teacher level, which verifies that teachers who feel obliged by the school or education authority to carry out professional development activities complete a significantly higher number of activities than those who do not perceive this obligation. Even though this result appears to be realistic, it is important to highlight that these significant differences are associated with an effect size with a low or very low relationship between both variables. Thus, despite greater professional development observed by teachers who feel obliged, this factor is not fundamental (it only explains 1.5% of the total variance in the number of training activities completed by teachers).

### 3.2. Decision Tree

Following a detailed analysis of the descriptive distribution of the number of training activities carried out by secondary teachers in Spain, the criterion variable of our study, we can now study the relationship between this factor and other educational and socio-demographic variables. Given the high number of predictor variables included in the study, data mining algorithms were used, specifically decision trees (Chen and Liu 2005; Martínez-Abad 2019; Quinlan 1986), which make it possible to find non-trivial information present in mass data sets (Martínez-Abad 2019). The sample was divided into two types of schools in order to obtain a decision tree that is easy to interpret:

- Schools with high levels of teacher training: schools in the top quartile of the study criterion variable (Xj > P75);
- Schools with low levels of teacher training: schools in the bottom quartile of the study criterion variable (Xj < P25).

A tree was obtained with 21 branches and 15 leaves, with 9 different variables. Overall, as regards precision levels, the true positives rate and areas under ROC and PRC curves of the tree obtain acceptable values in the training sample model, as seen in Table 4 (Zhou and Chen 2018). On the other hand, Kappa index (.499) and relative error RRSE (83.06%) values are above the desired level. Focusing on precision levels according to the type of school (with high or low teacher training), the model predicts schools in which teachers have low levels of training better than school with high levels of professional development. The loss of fit in the validated model is minor, maintaining the trends indicated above. Therefore, the model obtained can generally be deemed acceptable.

**Figure 3:**
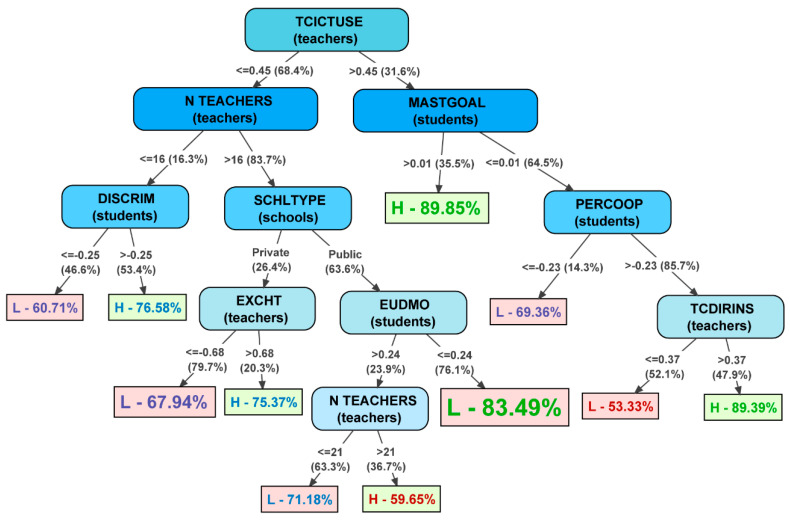
Decision tree.

The decision tree model obtained is shown in Figure 3. It represents the following elements:

- *Nodes:* Ellipses included in the tree present segmentation variables in descending order, from the variable with the highest power to explain teacher training level in the school (in this case, TCICTUSE), to the least important segmentation variables on the lower branches. Each node includes information on the segmentation variable and which PISA database it comes from.
- *Leaves (terminal nodes):* All paths on the tree descend to a rectangle or terminal node, also known as a leaf. Leaves include the following graphic information:
  - *Rectangle size and text font:* a bigger rectangle and font size indicate that the number of schools that reach this leaf is higher than smaller rectangles.
  - *Letter:* The letter in the leaf will be L if the sub-sample of schools on that path is associated with schools with low teacher training. The letter will be H if the path predicts schools with high training. Similarly, the colour of the rectangle also indicates whether the rule associated with the path predicts schools with high (green) or low (red) training.
  - *Percentage:* The percentage indicates precision in the prediction for schools that have reached this leaf. Over 80% indicates a high-precision rule; under 60% is a low-precision rule. Visually, the quality of the precision of each path is represented by the text colour: green of good precision, purple for acceptable, and red for low.
- *Branches:* The arrows between the nodes are the tree branches. The score shown in the arrows indicates the segmentation value of the sample in the variable of the previous node and, in brackets, the % of schools included in the previous node that follow this branch.

Of all the predictor variables included in the analysis, the following were maintained in the decision tree model:

- *SCHLTYPE (school):* Categorical variable related to ‘School Ownership’. According to ownership, there are three types of school in Spain:
  - *Privately managed schools*, which are in turn divided into two types: *schools with private ownership and funding*, and *privately owned schools with joint public–private funding*.
  - *Public schools*: publicly owned and funded.
- *TCICTUSE (teacher):* Aggregate composite variable from the teacher database, related to teachers’ use of specific ICT applications.
- *N TEACHERS (teacher):* Number of teachers at the school completing PISA surveys.
- *EXCHT (teacher):* Aggregate composite variable from the teacher database. Refers to teachers’ perception of exchange and coordination for teaching in the school.
- *TCDIRINS (teacher):* Aggregate composite variable from the teacher database. Assesses teachers’ perception of their own direct teachers’ instruction in the classroom.
- *DISCRIM (student):* Aggregate composite variable from the student database. Refers to students’ perception of discriminating school climate.
- *EUDMO (student):* Aggregate composite variable from the student database. Assesses teachers’ perception of their own eudaimonia *(meaning of life).*
- *MASTGOAL (student):* Aggregate composite variable from the student database. Assesses the student’s own level of mastery goal orientation.
- *PERCOOP (student):* Aggregate composite variable from the student database. Shows students’ perception of climate of cooperation at school.

Therefore, most variables included in the decision tree can be grouped around the factors presented in the literature review:

- *Innovation and development of quality educational practices: TCICTUSE and TCDIRINS;*
- *Distributed and shared leadership: EXCHT and PERCOOP;*
- *Collaboration culture and school climate: DISCRIM, MASTGOAL and EUDMO.*

It is important to point out that, while the significance of socio-demographic and economic factors in the decision tree obtained is marginal (N TEACHERS and SCHLTYPE), cognitive and meta-cognitive factors associated with students—such as skills in mathematics, reading and sciences—are not even indicated in the tree as relevant factors associated with high levels of professional teacher development.

Overall, the model presented in the tree highlights that schools with high levels of teacher development are associated with higher levels in the following factors: innovation, leadership and collaboration culture. This is supported by a detailed analysis of the main paths on the tree:

- *The two paths associated with high levels of training with greater precision include schools with high use of ICTs by teachers (TCICTUSE). The high level of training in these schools is fundamentally associated with students with high levels of orientation toward academic achievement (MASTGOAL). These schools that do not attain such high levels of academic achievement are also associated with high training if the students perceive reasonable levels of cooperation among the school community and if teachers implement adequate direct instruction.*
- *The main path associated with low levels of training has a precision of 83.49%. These are schools with low ICT use by teachers (TCICTUSE), a more complex and less controllable organisation (larger—NTEACHERS—and publicly owned—SCHLTYPE—schools), and students who are more pessimistic or concerned with the meaning of life and their own existence (EUDMO).*

## 4. Discussion and Conclusions

The main goal of this paper was to analyse the professional development of secondary teachers in Spain and detect key associated school factors. Taking into account the evidence obtained and presented, we can state that the results respond to this objective satisfactorily.

Firstly, focusing on RQ1 on the ongoing training topics teachers show more interest in, there is clear trend toward training activities related to educational technology and the development of specific and cross-cutting skills in students. These results are consistent with efforts by national and supranational institutions in relation to implementing training based on key skills (European Commission 2018; González and Wagenaar 2003; Halász and Michel 2011; Strijbos et al. 2015) and developing digital skills (Carretero et al. 2017; INTEF 2017; UNESCO 2018) in the Spanish education system. The topics most present in ongoing teacher training are associated with both educational innovation using ICTs and specific methodologies in the teacher’s specialist subject. However, teachers’ interest in ongoing training on other key areas such as educational leadership, effective communication, coordination with students and other teachers or classroom climate is much more marginal. This result contrasts with the importance attributed in the literature review to these factors in the development of smart schools (Aderet-German and Ben-Peretz 2020; Malagón and Graell 2022; Ortega-Rodríguez and Pozuelos 2022).

As for RQ2, the results clearly show a situation of uneven importance in relation to the commitment of secondary teachers in Spain to their own professional development. The high variability of this variable at teacher level highlights that, although a significant number of teachers are firmly committed to ongoing training, another considerable group does not refresh their teaching practices with regular training. This lack of commitment appears to stem more from the teacher’s personal sphere rather than the school, as teachers who are highly active and inactive in their professional development are evenly spread among schools. This issue is obviously an important handicap for achieving the goal of developing smart schools. This situation may be related to a lack of engagement with the teacher identity, associated with a traditional view of teacher training models (Hernández-Ramos et al. 2021). It should be noted that traditional models of teacher education gave more importance to initial than to in-service training. Another factor that may explain this evidence is the teacher transfer system in Spain (Martínez-Abad et al. 2019), which rewards professional development for non-pedagogical issues (e.g., obtaining points for transfers).

Finally, under RQ3, evidence was collected on the association between professional teacher development and correct implementation of shared leadership, cooperation and educational innovation factors in schools. The results obtained show that both matters are related. Of all the variables included in the initial models, the decision tree essentially included variables that can be associated with three key factors in the correct functioning of smart schools: schools in which teachers are generally more involved in their ongoing training are more innovative (more frequent use of ICTs a teaching tool for learning), have a more developed collaboration culture with distributed responsibilities (teachers cooperate with their colleagues more intensely in developing and achieving shared educational goals, and both teachers and students are more clearly oriented toward academic development) and have a more convenient school climate (more reduced discriminatory climate and students perceive a more appropriate exchange between members of the education community). It is important to once again note that, in line with previous studies (Aderet-German and Ben-Peretz 2020; Hargreaves 2003; Malagón and Graell 2022; Ortega-Rodríguez and Pozuelos 2022; Prenger et al. 2021), professional teacher development is more closely associated with these factors than with other factors that could initially be considered key: socio-demographic characteristics of schools and teachers, and student academic performance.

To conclude, the findings emphasise the importance of fostering ongoing teacher training, though not in general way, in the pursuit of specific purposes instead:

- On the one hand, in line with authors such as García-Garnica and Caballero (2019) or Bolivar et al. (2017), training actions should be developed and promoted that allow school members to act as pedagogical leaders under a distributed leadership philosophy, where both school and education community benefit and those teachers properly develop their professional teaching identity (Hernández-Ramos et al. 2021).
- Develop initiatives to improve coexistence and multi-way communication in the school (Torrecilla Sánchez et al. 2014), improving relationship between all members of the education community (students, parents and teachers). As shown in this paper, developing smart schools requires members to feel comfortable with a non-discriminatory climate, promoting cooperation and clear goals, such that all members fully understand the purpose of their actions.
- Incorporate technology in a planned, thought-out way based on suitably planned teaching innovations (Rodríguez-Conde et al. 2016). The teacher must be trained to incorporate technology resources in the classroom, though not with general training, with specific purposes instead. Moreover, the importance of collaborative incorporation must be stressed, involving students and other teachers in their innovations.

Given the non-experimental and secondary data analysis nature of this research, it suffers from one important weakness that should be addressed in future, more specific studies dealing with a greater number of variables. Firstly, the study criterion variable was created based on teachers’ statements on subjects and aspects in which they have received ongoing training. There may be bias associated with these inferences as this variable was assimilated to the quantity of training received by teachers. Moreover, PISA 2018 databases did not include some key factors related to professional teacher identity or educational assessment, which could have shed more light and certainty on the results obtained. Both limitations must be addressed in future replication studies that include these scales specifically in samples obtained ad hoc. It is also important to note that this study is correlational, making it very difficult to establish a cause–effect relationship. In this regard, it is essential to propose a longitudinal research approach in future work, ideally with experimental control, in order to verify the hypotheses arising from this paper. Finally, these data were collected by the OECD in 2018 before the COVID-19 global pandemic. Given the profound transformation caused by the pandemic in education systems worldwide, it is highly likely that both priority teacher training areas and innovation, leadership and cooperation culture processes in schools have undergone a significant evolution. Therefore, future research must replicate this paper with updated data, using PISA 2022 assessments, for example, which are likely to be available to the academic community from December 2023.

## Figures and Tables

**Table 1:** Explanatory variables included in the study.

Name	Tag	Database
REPEAT	Grade Repetition	Student
BSMJ	Student’s expected occupational status	Student
TMINS	Learning time (minutes per week)—in total	Student
ESCS	Index of economic, social and cultural status	Student
UNDREM	Meta-cognition: understanding and remembering	Student
METASUM	Meta-cognition: summarising	Student
METASPAM	Meta-cognition: assess credibility	Student
DISCLIMA	Disciplinary climate in test language lessons	Student
DIRINS	Teacher-directed instruction	Student
PERFEED	Perceived feedback	Student
STIMREAD	Teacher’s stimulation of reading engagement perceived by student	Student
ADAPTIVITY	Adaptation of instruction	Student
TEACHINT	Perceived teacher’s interest	Student
JOYREAD	Joy/Like reading	Student
PERCOMP	Perception of competitiveness at school	Student
PERCOOP	Perception of cooperation at school	Student
ATTLNACT	Attitude towards school: learning activities	Student
COMPETE	Competitiveness	Student
WORKMAST	Work mastery	Student
GFOFAIL	General fear of failure	Student
EUDMO	Eudaemonia: meaning in life	Student
SWBP	Subjective well-being: positive affect	Student
RESILIENCE	Resilience	Student
MASTGOAL	Mastery goal orientation	Student
DISCRIM	Discriminating school climate	Student
BELONG	Subjective well-being: Sense of belonging to school	Student
BEINGBULLIED	Student’s experience of being bullied	Student
USESCH	Use of ICT at school in general	Student
INTICT	Interest in ICT	Student
COMPICT	Perceived ICT competence	Student
AUTICT	Perceived autonomy related to ICT use	Student
ICTCLASS	Subject-related ICT use during lessons	Student
PV10MATH	Plausible Value 10 in Mathematics	Student
PV10READ	Plausible Value 10 in Reading	Student
PV10SCIE	Plausible Value 10 in Science	Student
EMPLTIM	Teacher employment time—dichotomous	Teacher
TCSTAFFSHORT	Teacher’s view on staff shortage	Teacher
EXCHT	Exchange and co-ordination for teaching	Teacher
SATJOB	Teacher’s satisfaction with the current job environment	Teacher
SATTEACH	Teacher’s satisfaction with teaching profession	Teacher
SEFFCM	Teacher’s self-efficacy in classroom management	Teacher
SEFFREL	Teacher’s self-efficacy in maintaining positive relations with students	Teacher
SEFFINS	Teacher’s self-efficacy in instructional settings	Teacher
TCICTUSE	Teacher’s use of specific ICT applications	Teacher
TCDIRINS	Direct teacher’s instruction	Teacher
FEEDBACK	Feedback provided by the teachers	Teacher
ADAPTINSTR	Student assessment/use (adaption of instruction)	Teacher
FEEDBINSTR	Feedback provided by the teachers	Teacher
SC001Q01TA	Which of the following definitions best describes the community in which your school is located?	School
SCHLTYPE	School ownership	School
STRATIO	Student–teacher ratio	School
SCHSIZE	School size	School
STAFFSHORT	Shortage of educational staff	School
STUBEHA	Student behaviour hindering learning	School
TEACHBEHA	Teacher behaviour hindering learning	School

**Table 2:** Descriptive statistics of the number of professional development activities.

	Mean	*S_x_*	Min.	P25	P50	P75	Max.
Teacher level	4995	4892	0	1000	4000	8000	14,000
School level	5055	1630	0	3947	4909	6000	18,000

**Table 3:** Relationship between the obligation to complete professional development and frequency.

	Descriptive		U Mann–Whitney *			
	Mean	*S_x_*	Z	*p*	r_bp_	η^2^
Yes	5.52	4.97	−18.35	<.001	.155	.015
No	4.32	4.69				

* The nonparametric contrast is applied for two independent groups because the normality assumption for the criterion variable is not met (Figure 2).

**Table 4:** Precision model obtained in the decision tree.

		TP	Prec.	PR	ROC
Training set	Low training	.897	.750	.808	.806
	High training	.583	.803	.749	.806
	Global fit	.766	.772	.783	.806
Cross-Validation	Low training	.748	.668	.714	.669
	High training	.481	.578	.589	.669
	Global fit	.636	.630	.662	.669

## Data Availability

PISA 2018 data are freely accessible from the OECD website: https://www.oecd.org/pisa/data/2018database/.

## Note

1 In PISA, composite factors refer to variables obtained from the aggregation of a set of items. For example, the scores for each student on the JOYREAD (Joy/Like reading) composite factor are obtained from the student’s responses to several items that relate to liking to read. Thus, in PISA Students database is available a composite variable called JOYREAD.
